# *In vitro* larval rearing protocol for the stingless bee species *Melipona scutellaris* for toxicological studies

**DOI:** 10.1371/journal.pone.0213109

**Published:** 2019-03-20

**Authors:** Adna Suelen Dorigo, Annelise de Souza Rosa-Fontana, Hellen Maria Soares-Lima, Juliana Stephanie Galaschi-Teixeira, Roberta Cornélio Ferreira Nocelli, Osmar Malaspina

**Affiliations:** 1 Centro de Estudos de Insetos Sociais—CEIS, Instituto de Biociências, Universidade Estadual Paulista Júlio de Mesquita Filho (UNESP-SP), Rio Claro, SP, Brasil; 2 Instituto Tecnológico Vale–Desenvolvimento Sustentável (ITVDS), Instituto Tecnológico Vale—Belém II, Belém, PA, Brasil; 3 Departamento de Ciências da Natureza, Matemática e Educação, Universidade Federal de São Carlos (UFSCar-SP), Araras, SP, Brasil; University of California San Diego, UNITED STATES

## Abstract

Brazil has the highest biodiversity of native stingless bees in the world. However, Brazilian regulations are based on protocols standardized by the Organization for Economic Cooperation and Development (OECD), which uses *Apis mellifera* as a model organism. The safety of the use of an exotic species as a substitute for a native species is a problem that concerns members of the academy and the government agencies responsible for studies of this nature in the neotropical regions where there are occurrences of stingless bee species. Regarding the exposure of larvae to pesticides, several indicators suggest that the same rearing method for *A*. *mellifera* cannot be applied to stingless bees, mainly because of their different feeding systems. Thus, it is necessary to establish an *in vitro* rearing method for native social bees. We developed a larval rearing method for the stingless bee species *Melipona scutellaris* and evaluated parameters such as the defecation rate, pupation, emergence, mortality and morphometry of the newly emerged workers. The control was represented by the morphometry of individuals that emerged from natural combs (*in vivo*). In addition, we determined the average lethal concentration (LC_50_) of the insecticide dimethoate, the standard active ingredient used for the validation of toxicity tests. Procedures conducted prior to the *in vitro* bioassays allowed us to obtain the actual dimensions of the rearing cells for making acrylic plates for use in establishing how much each larva consumes during its development, that is, determining how much larval food should be placed in every artificial cell. Tests performed with *M*. *scutellaris* indicated an average of 80.2% emergence of individuals relative to the larvae, 92.61% relative to the pupae and a mean of 7.42% larval mortality. The mean of the intertegular distance, head width and wing asymmetry parameters were not significantly different between individuals from the *in vitro* and *in vivo* rearing methods. The LC_50_ value determined was 27.48 ng dimethoate / μL diet. The method described for *M*. *scutellaris* showed development rates above OECD standards, which requires at least 75% emergence, and produced newly emerged workers with similar dimensions to those produced under natural conditions; thus these results enable their use as a rearing protocol for this species (or genus) and, consequently, their use in toxicity tests. The results produced with *M*. *scutellaris* are the first steps for a proposed toxicity test protocol for stingless bee larvae that can be standardized and included as a protocol in the OECD.

## Introduction

It is estimated that there are more than four thousand genera and approximately 20 thousand species of bees distributed in different regions of the world [1]. Brazil, due to its continental proportions and rich ecosystems, has approximately 5,000 of these species distributed in five families [2]. The stingless bees (Apidae: Meliponini) are the largest group of eusocial bees in the world, occur in the neotropical region and live in perennial colonies that can range from tens to thousands of individuals [1]. Of the native stingless bees, there are currently 244 valid species and approximately 89 species not yet described in 29 genera (excluding extinct groups) [3].

This group shares highly social (eusocial) habits with the Apini tribe (which includes the species *Apis mellifera*). Their general habit makes them visit a wide range of floral resources, being that way essential pollinators of native plants [4] and important for agricultural crops [5]. In Brazil, of 141 crops, 85 depend on pollinators, including stingless bees, and pollination services generate a market of approximately $12 billion annually [6]. In addition, stingless bees pollinate approximately 66% of the world's 1,500 crop species, accounting for 15–30% of world food production [7].

However, such ecosystem services provided by these pollinators have diminished with the increasing decline of pollinators, which consequently generates negative impacts, such as the loss of the economic value of the crops, which can lead to a significant social impact due to the lack of food and to the decrease and extinction of several plant species [8; 9]. Crop production effects can be mitigated with managed pollinators [6]. Several causes have been attributed to the decline of pollinators, such as climate change, habitat loss, excessive use of pesticides and, above all, the interactions among these factors.

Bees are subject to the action of pesticides by the body contact, ingestion of pollen and nectar contaminated when spraying during the period of flowering, or by the ingestion of contaminated floral resources through the systemic action of the active ingredients of the neonicotinoids [10].

The data on the toxicity of agrochemicals on bees use the species *A*. *mellifera* (in both immature and adult stages) since it has spread globally and is considered the main pollinator of many agricultural crops that feed the world [11]. Most of the works on stingless bees were carried out with adult individuals [12,13,14,15,16,17,18].

As well as in *A*. *mellifera*, due to the eusocial habit of stingless bees, in addition to foraging, they come in direct contact with pesticides in the field. When they return to the hive, they can carry potentially contaminated floral resources with residues that will later be processed by the nurse workers and offered to the brood [10,19]. Therefore, it is important to emphasize that the different stages of development of these pollinators are relevant for evaluation, which includes exposure during the larval phase through ingestion of residues [20].

The method for the larval rearing of *A*. *mellifera* is already standardized and recognized by the Organization for Economic Cooperation and Development [21] based on the work of Aupinel et al. [22] and Aupinel et al. [23]. These protocols were adopted by the United States environmental agency for the schematization of risk assessment, which was adopted as a reference for the current risk assessment scheme in Brazil. However, among the main uncertainties noted by members of the academy and government agencies responsible for studies of this nature in the neotropical region (IBAMA in Brazil), where there are occurrences of species of stingless bees, the use of honey bees as model organisms stands out, since stingless bees are part of the native pollinator fauna. One way of remedying this doubt would be to carry out a comparison of *A*. *mellifera* with native species regarding the exposure levels and the toxicity of pesticides.

The development of methods for studies with larvae allow for a better evaluation and representation of what happens in the colony with the larvae that are exposed to pesticides brought by the workers. However, the need for the development and standardization of *in vitro* breeding methods for stingless bees is emphasized, since larval feeding systems differ between groups; in *A*. *mellifera*, the nurse bees progressively deposit food to the offspring [24], but in stingless bees, they do it all at once, depositing all of the food that will be consumed [19, 25].

The first method of stingless bee rearing was described by Camargo [26], but this presented some lacks that made it impossible to obtain newly emerged individuals in sufficient numbers to perform toxicity tests. In recent years, a study by Menezes et al. [27] presented adaptations to the method described by Camargo et al. [26] and applied them to the *in vitro* rearing of *Scaptotrigona depilis* queens. The results showed a good emergence rate when adjustments in relative air humidity and feed quantity were made during the experiment, and the bees that emerged from the experiment were characterized as similar to naturally emergent bees, which opened up new possibilities for tests with stingless bees. However, there is not yet a standardized method that caters to stingless bees.

For adequately planning public policies to protect bees in Brazil, there is a need to establish a species representative of native bee fauna to be considered a model organism as well as the standardization of methods for the execution of the toxicity of these organisms. In addition, according to IBAMA [28], among the main limitations for pesticide risk assessments for native bees is the lack of basic data on the biology of these bees. In this context, Dorigo et al. [29] provided data such as food consumption at different life stages and the mass of the individuals of three stingless bee species, one of them being *M*. *scutellaris*. This native bee in Brazil is one of the priority social bee species for pesticide risk assessment according to the recent list of species selection, which considered several criteria, such as geographic distribution, association with agricultural crops, and importance as pollinators, among others [30]. For these reasons, it is a species with the potential to be considered a model organism in risk assessments. The standardization of an *in vitro* rearing method for stingless bees can simulate the actual effects of these chemical compounds on bees, contributing to the construction of regulatory actions that assist in the conservation of native pollinators.

Due to the current demands, we aim to establish an *in vitro* breeding method for *M*. *scutellaris* so that the proposed protocol can be used in toxicological studies with stingless bees.

## Materials and methods

Research on invertebrates does not require animal ethics approval in Brazil.

### Study place, species chosen and ideal colony conditions for sample collection

The combs containing *M*. *scutellaris* larvae were collected in the meliponary of the Institute of Biosciences, Department of Biology of the "Paulista Júlio de Mesquita Filho State University", campus of Rio Claro; three unrelated parental colonies were used for experiments, and the ideal conditions for collection included being of the same health, not having diseases or parasites, having a great number of individuals and having young and strong queens, which can be identified by the coloring of the abdomen and the wear of its wings. The collection of the combs was performed in a way that did not harm the sanity of the colonies, and the boxes were opened with care so as not to damage the honey pots and pollen (S1 Video).

### Procedures preceding *in vitro* bioassays

**Fig 1:**
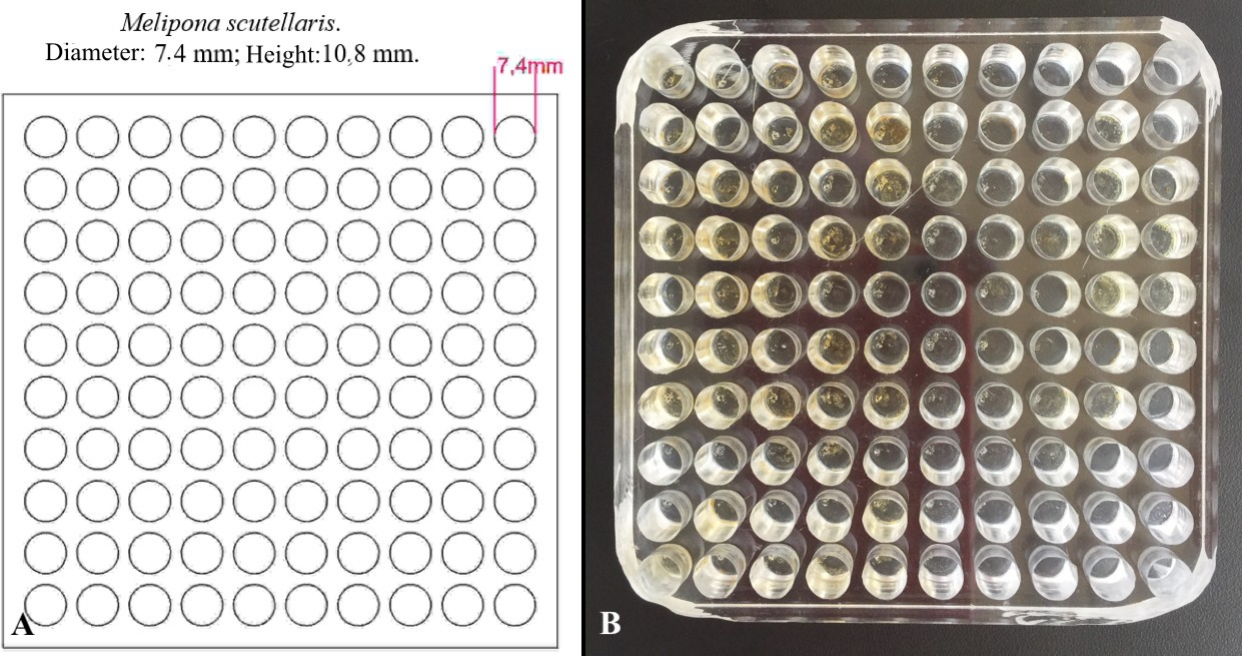
Acrylic plate used for the larval bioassays. The plate’s measures are in to the figure.

To measure the amount of larval food per brood cell, 20 randomly chosen cells per colony were collected. The collected combs were already operculated and contained eggs that had just been oviposited. With an automatic micropipette, the food from each colony was homogenized in Eppendorf microtubes with volumetric graduation (obtaining the total volume). The Eppendorf microtubes were weighed on an analytical balance before and after food collection (obtaining the total mass). The volume and total mass were divided by the number of brood cells (n = 60), estimating the amount of individual food per cell. In parallel, the height and diameter of the natural comb cells were measured using a digital caliper to reproduce the cell dimensions in acrylic plates for the experiments, as shown in Fig 1.

### Description of the proposed protocol

From the average volume of food that the larvae consume and the production of reproduced acrylic plates, new samplings were carried out for the beginning of the *in vitro* experiments, distributing the amount of food pre-established, by brood cell. The technique adopted for the bioassays was based on the method developed by Menezes et al. [27] for the rearing of *S*. *depilis* queens, adapting the technique for the development of *M*. *scutellaris* workers.

During the handling of the combs to remove food and larvae, the room was maintained at a humidity above 75% to prevent the food, eggs and larvae from dehydrating. For this purpose, a container of water was boiled using two electric boilers along with an air humidifier. Prior to the transfer of food and larvae, the 100-well acrylic plates were washed, sterilized and packed in capped glass Petri dishes (1.5 x 20 cm) with the bottom filled with distilled water. The room environment modified with boiling water and the water within the plates are required just during the first 5 days of incubation, in order to keep the humidity above 75% and between 95 ± 5%, respectively. The brood combs were collected from the colonies and taken to the laboratory. Two types of brood comb were collected, one containing eggs for food withdrawal and the other containing first instar larvae (S2 Video). The cells containing eggs were desoperculted and the eggs were removed. The transport of the combs to the wet room was carried out inside Petri dishes with cotton moistened with water.

**Fig 2:**
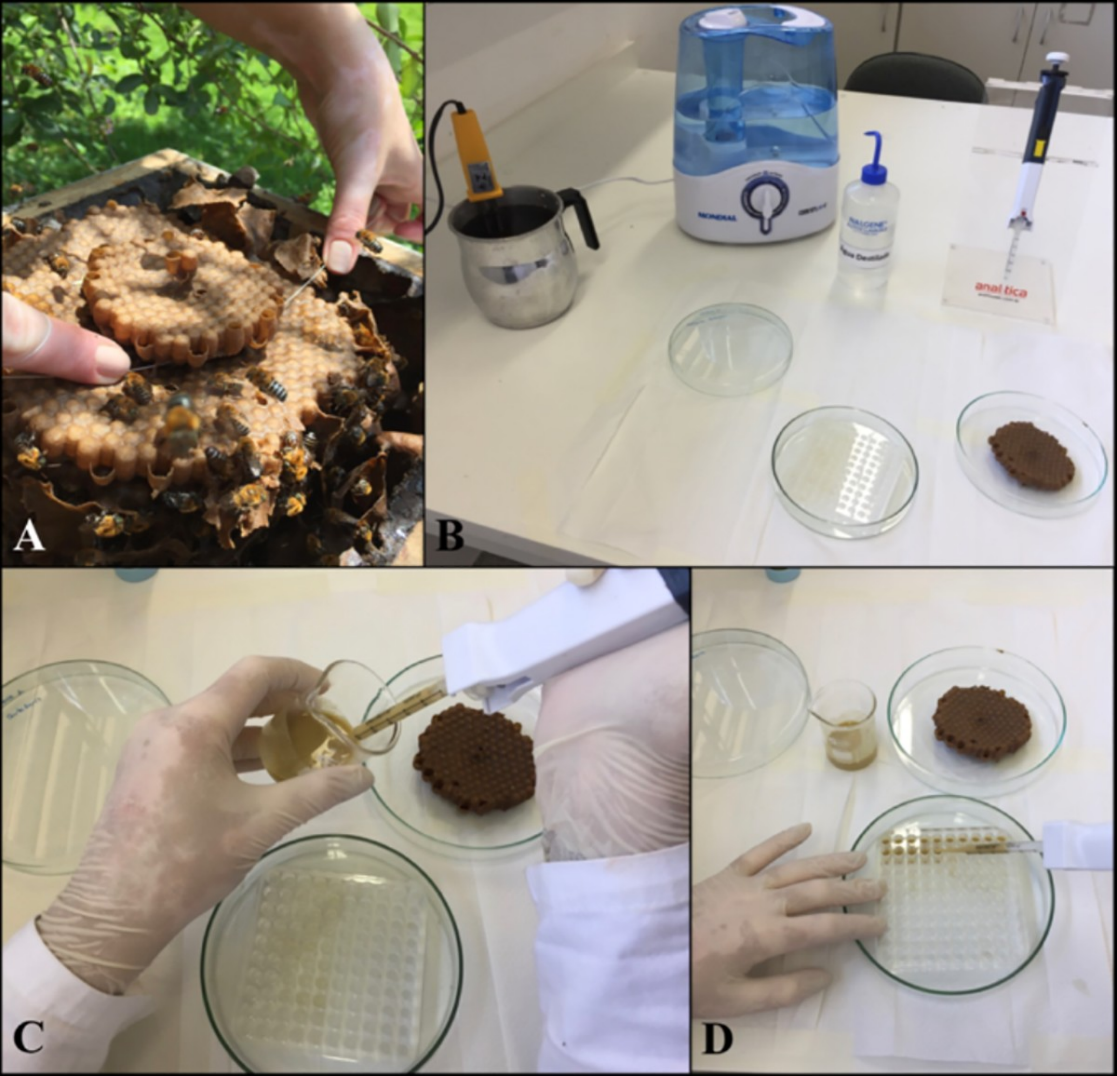
Representation of the experimental phases of the *in vitro* rearing of bees of the *Melipona scutellaris* species. (A) Collection of combs from the colonies. (B) Preparing the environment of the room for transference. (C) and (D) Food distribution on the acrylic plates.

**Fig 3:**
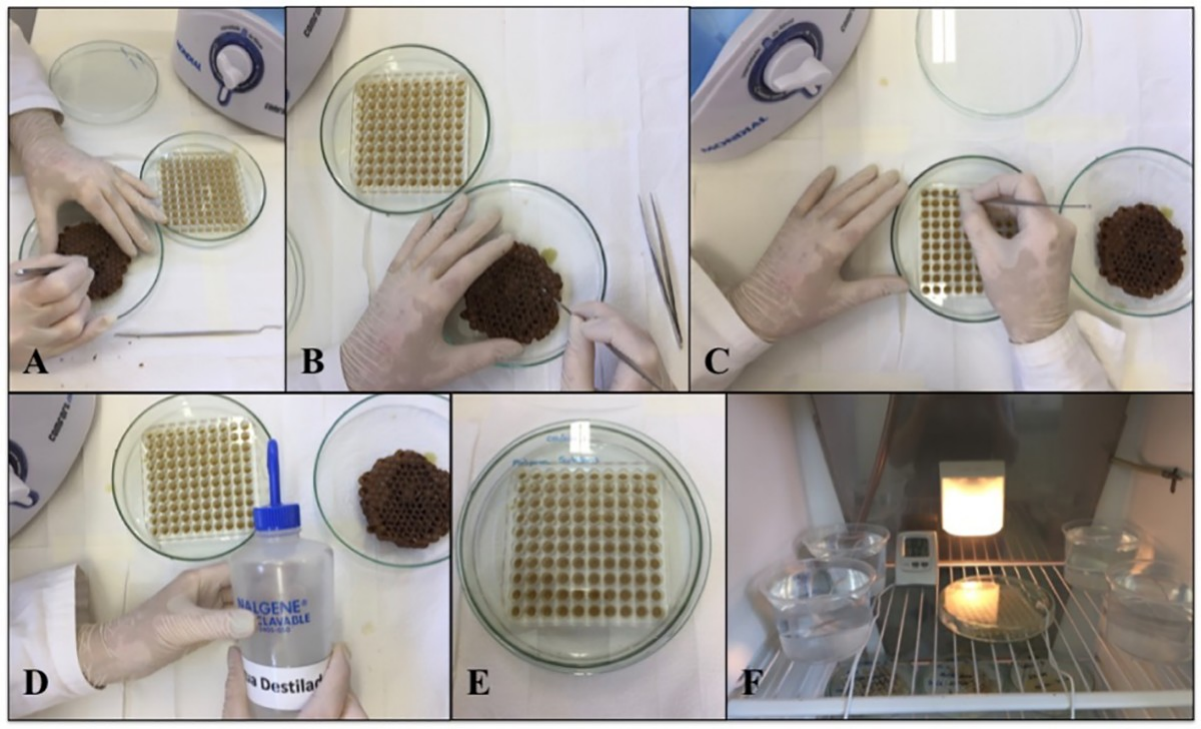
Representation of the experimental phases of the *in vitro* rearing of bees of the *Melipona scutellaris* species. (A) Desoperculation of the comb. (B) and (C) Larval tranference. (D) Addition of distilled water to Petri dish. (E) Transferred larvae. (F) Experiment in progress.

**Fig 4:**
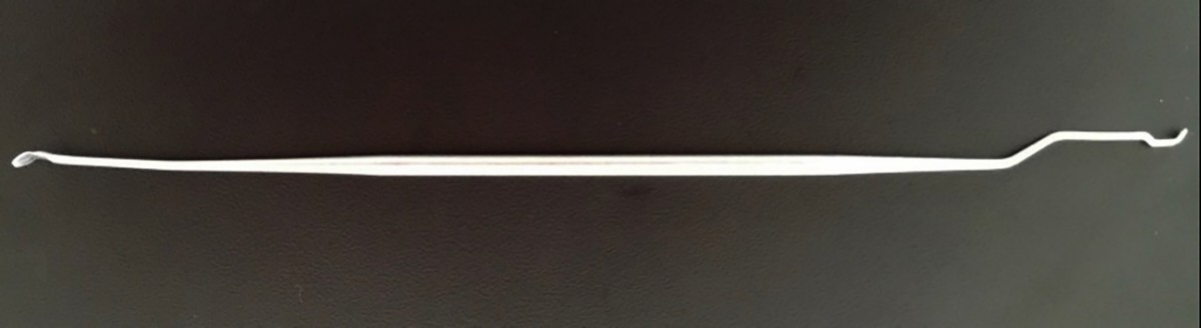
Bee needle used in larval transference.

Then, the larval food was collected with an automatic micropipette, and 13 mL of food from each colony was collected, totaling 39 mL. Then, all the collected food was mixed and homogenized to be distributed through the plates with a repetitive micropipette, with 130 μL of larval food deposited inside each well of the acrylic plate, and soon after, the larvae were transferred with the help of a bee needle, as shown in Figs 2, 3 and 4. The time between placing the food in a cell, up to the plate to be placed in the incubator lasts about 10 minutes.

During larval transference, it is not possible to distinguish the brood cells in which the workers, queens or males will emerge. After the transfer, the Petri dishes with distilled water and incubated in a BOD incubator (Biochemical oxygen demand) at 30°C and 95% relative humidity. The temperature conditions were maintained until the end of the experiment, but the humidity conditions of BOD were altered during development.

After 5 days of incubation, or when the total food consumption by the larvae was noted, the humidity inside the plates was reduced to 75%, and a solution of sodium chloride (NaCl) was added to the Petri dish. During all of the period of the experiments, temperature and relative humidity conditions were controlled with a thermohygrometer.

The experiments were repeated four times at intervals that did not affect the development of the colonies (approximately 30 days); in experiment, 3 plates were used, totaling the transfer of 1.200 larvae. To evaluate the feasibility of the use of eggs *in vitro*, a fourth plate was also added in the first two experiments, also having 3 replicates and each containing 20 transferred eggs, totaling 120 eggs. We used a low number of individuals because some previous experiments (results not included herein) had already shown high mortality.

### Parameters used for method validation

Several parameters that are indicative of the progression of the larval stages of development such as: feeding; defecation; pupation; and emergence were observed and recorded daily after the transfer of the larvae to verify the functionality of the method. Thus, it was possible to compare our results with the mortality/emergence rates already obtained by Aupinel et al. [22], which are the larval mortality values already recognized by the OECD [21]. Mortality and emergence calculations were included non-worker caste, but, for both developmental time and morphometrical analysis males and queens were excluded from these analyses, and only workers were used. Visually, it is possible to differentiate among the castes when the pupae begin the process of body’s pigmentation. Dead individuals were withdrawn daily from the plates.

Morphometric parameters were analyzed for both *in vitro* and *in vivo* emerged bees to compare the similarity between them and to verify possible deformations from *in vitro* rearing. The same number of individuals were used for both of the experiments, the brood combs came from the same source colonies, at the same period, and we used the same procedure to remove and take them to the laboratory. The only difference was the age of the *in vivo* comb collected: instead of to collect brood containing newly emerged larvae, we took emergent combs and placed them inside BOD for 24 hours (until the emergence).

**Fig 5:**
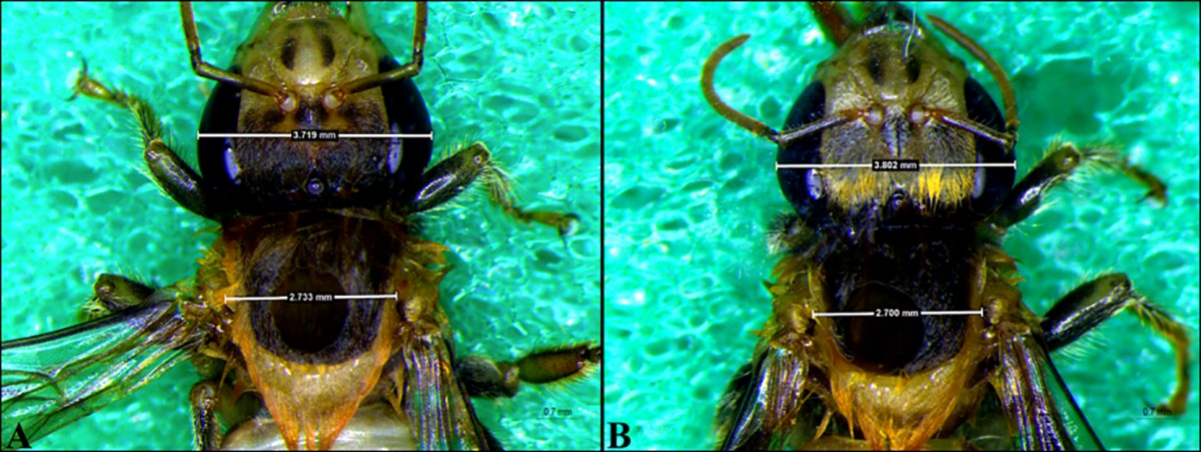
Head width and intertegular distance of *Melipona scutellaris* workers reared *in vitro* and *in vivo*. (A) Bee emerged *in vitro*; (B) Bees emerged *in vivo*. Values within the rectangles indicate the means of the morphometric measurements.

For intertegular distance and head width (Fig 5A and 5B) analysis, 10 bees per colony were used, totaling 30 individuals per experiment. The measurements were made by using images obtained with a stereomicroscope with a coupled camera (Leica M 205 C) and LAS V4.8 software using the measurement module of the software itself.

**Fig 6:**
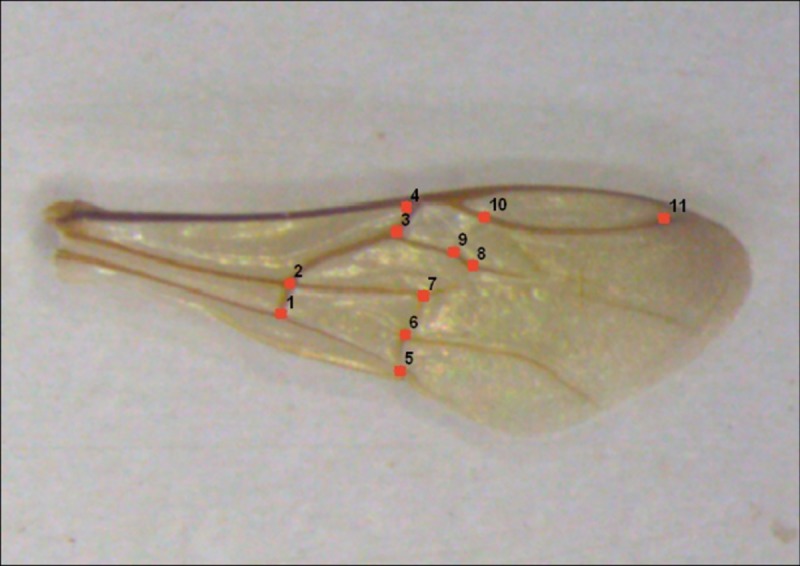
Anatomical marks of the joints of the wings of *Melipona scutellaris* workers with 24 hours of emergence.

Using the measures described above, possible asymmetries in the anterior wings of the bees emerged *in vitro* and *in vivo* were analyzed. For each procedure, we used the same 10 bees per colony describe above, totaling 30 individuals per experiment. The right and left anterior wings of the individuals were removed, mounted on slides, and photographed with a digital camera coupled to a stereomicroscope. From the photographs, a .tps file was built with the help of tpsUtil software version 1.40 [31]. Eleven anatomical landmarks were established (Fig 6) with software tpsDig version 2.12 [31], and parameters such as centroid size (square root of the sum of the square distances of the anatomical landmarks to the centroid or the center of mass) and wing shape from Procrustes residues [32] were assessed.

### LC_50_ determination of insecticide dimethoate

Procedures for the determination of the oral LC_50_ of dimethoate, the standard active ingredient used for the validation of toxicity tests for pesticides, were based on the OECD protocol [21]. For the bioassays, the insecticide was diluted directly into the larval food, and subsequent successive dilutions were carried out in the food to reach the concentrations to be offered to the larvae (in ng active ingredient / larva): 272, 136, 68, 34, 17, 8.5, 4.25 and 2.125. Since stingless bee larvae consume all the food deposited in the breeding cell, it is possible to determine the total ingested concentration. Each bioassay consisted of 3 replicates of 20 larvae in each concentration. The mortality of the individuals was recorded 24 hours after the end of the feeding (144 h after the transfer of the larvae).

The mortality of individuals was evaluated daily up to 48 hours after the end of feeding (168h of bioassay), with all dead individuals being withdrawn daily. The bioassays were not performed simultaneously and were performed with 20 larvae at each concentration in triplicate, totaling 540 larvae. The negative control was given by the larvae fed with pure larval feed, without addition of pesticide.

### Statistical analysis

The data concerning the emergence of the bees relative to the number of transferred larvae, the emergence of the bees relative to the number of pupae and the mortality of the larvae on the 5th day of experiment (after consumption of all food offered) were statistically analyzed using software R (R Core Team 2016). For each of the three parameters, association tests were performed between the different standardization bioassays. The probability of association (p-value) was obtained by a Pearson's chi-square or Fisher's exact test according to the composition of the analyzed data (sample size and expected frequency), and p <0.05 was considered significant. As multiple comparisons were performed, the p values were adjusted by the Benjamini-Hochberg false rate of discovery method (FDR).

To compare the averages of the intertegular distance and head width obtained in the *in vitro* and *in vivo* experiments, a t-test for independent samples was used (with a significance level of 0.05). A standard ANOVA for floating asymmetry was used for centroid size analysis [33], and a corresponding Procrustes ANOVA was used to analyze the shape of the wings [32]. In each analysis, the parameters "individual" (indicates comparison between the individuals without distinguishing the sides) and "side" (representing the right and left side of the same individual) were evaluated. These analyzes were conducted in MorphoJ v. 1.06d [34].

For analysis and the determination of the LC_50_, mortality data were observed every 24 hours and were statistically analyzed using software R (R Core Team 2016). For each observed time (from 24 to 168 hours), association tests were performed between the concentrations of dimethoate tested. The probability of association and adjustment were performed in the same way as the relative emergency and mortality rates described above.

The mean lethal concentration (LC_50_) values for dimethoate were determined with the 144 and 168 hour mortality data, which corresponded to 24 and 48 hours, respectively, after the total consumption of food contaminated with the insecticide by the larvae. The LC_50_ values as well as the 95% confidence interval and the chi-square values were established from the work of Pacheco and Rebelo [35].

## Results

### Development

**Fig 7:**
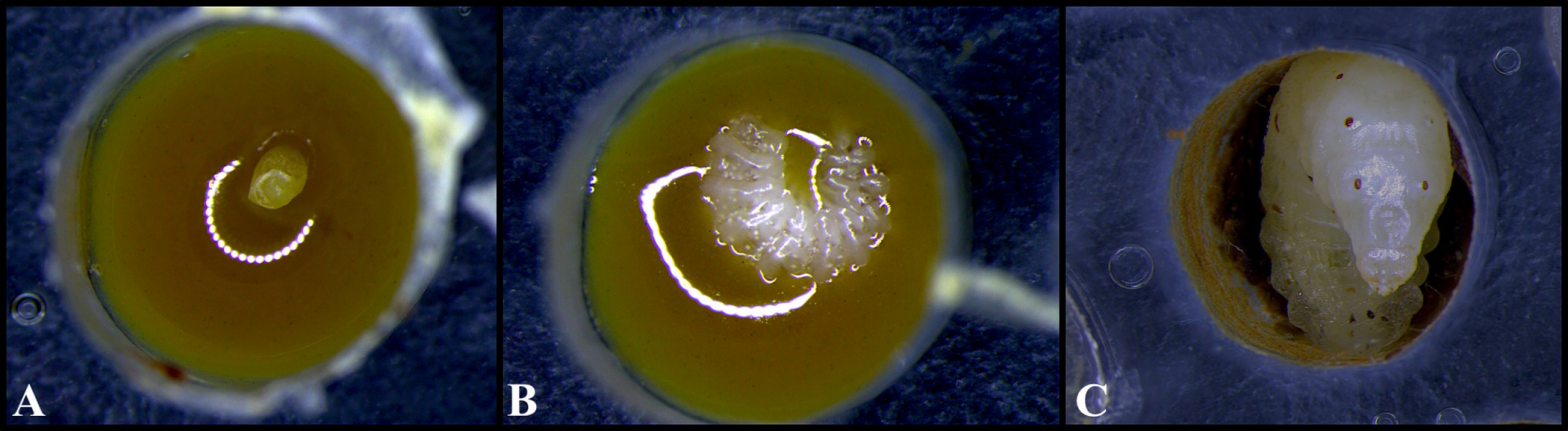
Egg end larval phases of the development of *Melipona scutellaris* workers. (A) Egg; (B) Larva 24 h after hatching and (C) Larva in 5^th^ day.

**Fig 8:**
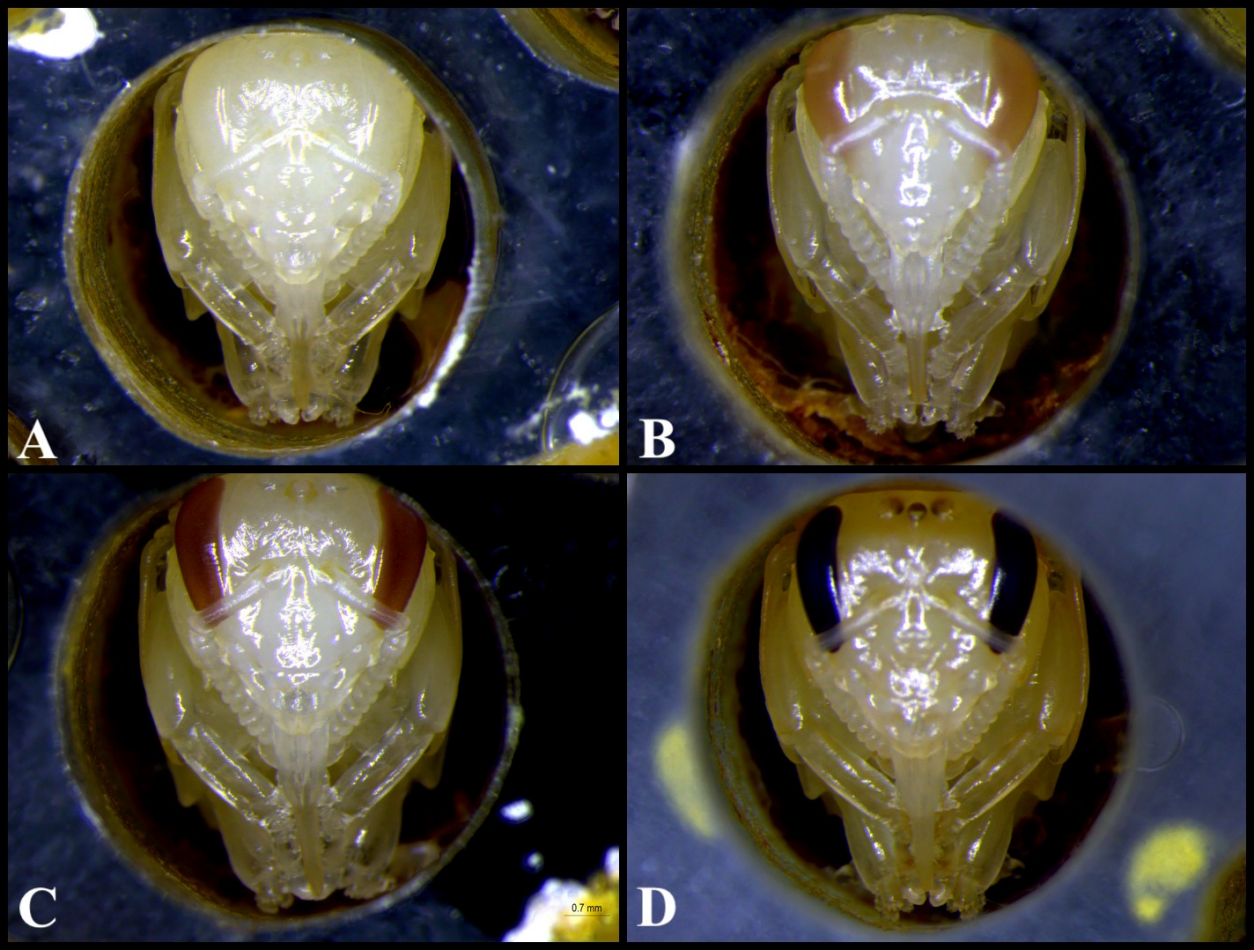
Phases of pupae from the development of *Melipona scutellaris* workers. (A) Pupa with white eye; (B) Pupa with pink eye; (C) Pupa with brown eye; (D) Pupa with black eye.

The tests performed with *M*. *scutellaris* indicated a mean of 80.2% (n = 755; ± 8.2) emergence of individuals relative to the larvae, 92.61% (n = 647; ± 5.48) relative to the pupae and a mean of 7.42% (n = 755; ± 4.95) larval mortality (Table 1). The comparative chi-square analysis showed that all of all the experiments were statistically similar (p> 0.05). Table 2 shows the events that occur during development of *M*. *scutellaris* workers, according to the proposed protocol. Each phase is represented in Figs 7 and 8. In relation to the two attempts of egg transfers, all of the eggs has died before hatching into larvae.

**Table 1:** Parameters validating the proposed rearing development protocol for *Melipona scutellaris*.

Experiment	Emergence/ larvae rate (%)*	Emergence/ pupae rate (%)^Ω^	Mortality/ larvae rate (%)^β^
1	77.87 ± 8.18	89.14 ± 3.19	10.72 ± 6.37
2	76.38 ± 6.13	94.05 ± 8.4	13.84 ± 3.05
3	78.88 ± 7.22	93.54 ± 3.48	8.26 ± 5.14
4	87.83 ± 9.64	93.72 ± 6.93	6.45 ± 3.55
Mean	**80.2 ± 8.2**	**92.61 ± 5.48**	**9.82 ± 4.95**
Mean	**69.7⊥**	**87.34⊥**	**≦ 15†**

* Emergence/ larvae rate: number of emerged bees -100/ initial sample number of larvae

^**Ω**^ emergence/ pupae rate: number of emerged bees -100/ sample number of pupae

^**β**^ mortality/ larvae rate: number of dead larvae on the 13^th^ day (last instar larval) -100/ initial sample number of larvae.

**⊥** Means obtained by Aupinel et al. (2005)

† Mortality rate indicated in TG 237 (OECD, 2013).

± indicates the values of the standard deviations of the means.

**Table 2:** Events occurring during development according to the proposed protocol for *Melipona scutellaris* workers.

Test	Duration of feeding (days)	Defecation	Pre-pupa	Pupa white eye*	Pupa pink eye*	Pupa brown eye*	Pupa black eye*	Duration to emergence (days)
**T1**	5	7°	13°	15°	18°	20°	24°	40
**T2**	4	7°	14°	16°	18°	20°	25°	38
**T3**	5	9°	14°	16°	22°	24°	27°	45
**T4**	5	5°	15°	17°	19°	21°	23°	43
**Mean**	4.8±0.45	7°±1.41	14°±0.81	15.8°±0.84	18°±1.74	21.2°±1.65	24.6°±1.5	41.4°±2.7

* The ordinal numbers indicate the exact days in which the events occurred.

± indicates the values of the standard deviations of the means.

### Morphometry

#### Intertegular distance and head width

**Fig 9:**
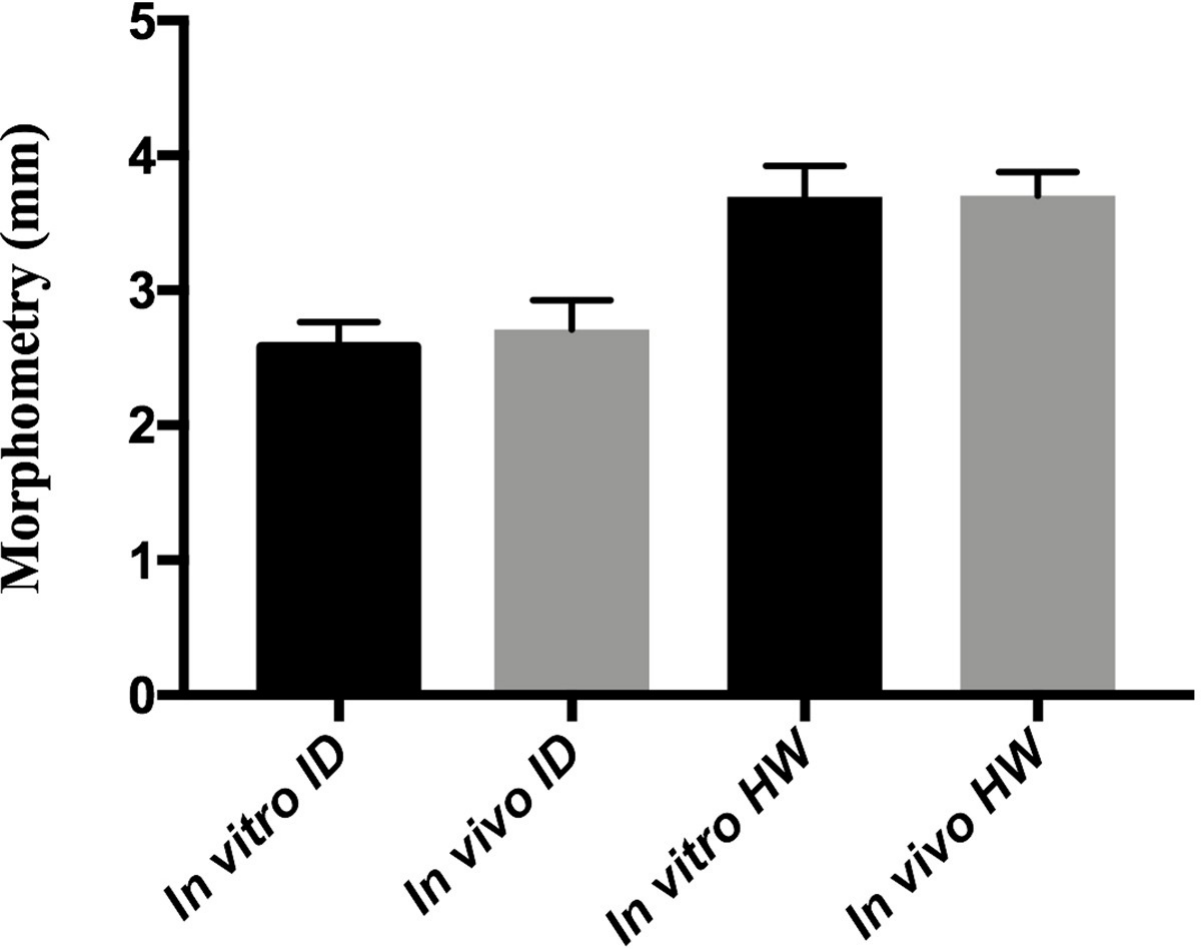
Morphometric measures (mean in millimeters) of *Melipona scutellaris* workers reared *in vitro* and *in vivo*. (ID—Intertegular distance; HW—Head width).

The mean of the intertegular distance for the *in vitro* method was 2.58 mm, and for the *in vivo* method, it was 2.67 mm. The head width was 3.68 mm *in vitro* and 3.70 mm *in vivo*. The two t tests carried out for each measure (intertegular distance and head width), indicated no significant difference between the *in vivo* and *in vitro* methods, both of them with p> 0.05 (Fig 9).

#### Wing asymmetry

The Procrustes ANOVA analysis indicated a significant difference in the wing venation pattern in the "individual" effect only in the individuals emerged from the *in vitro* rearing ("individual": F = 1.49, p <0.0001) (Table 3).

**Table 3:** Centroid size ANOVA and wing venation pattern of *Melipona scutellaris* workers emerged *in vitro* and *in vivo*.

Parameters	Effect	*In vitro*	*In vivo*
**Centroid size**^**1**^	Individual^a^	7816.9967	5833.106
	Side^b^	1943.2104	28047.354
**Wing venation pattern**^**1**^	Individual^a^ Side^b^	0.0003466* 0.0003991	0.0003894 0.0007536

*P<0.0005

^1^ The centroid size and wing venation pattern were analyzed by the standard ANOVA for floating asymmetry and the corresponding Procrustes ANOVA, respectively.

^a^ "Individual" indicates the comparison between individuals without distinction of the sides

^b^ "Side" represents the comparison between the left and right sides of the same individual; the numbers correspond to the values of the average squares.

#### LC_50_ of dimethoate

After 24 hours of the first bioassay, in which the insecticide was diluted in water and then added to the larval food, it was observed that the food content had been separated from the liquid part and was concentrated only in the lower part of the cell. Thus, the larvae had access only to the aqueous part, which caused 100% mortality in the transferred larvae because they fed on only the contaminated water accumulated in the upper part of the food.

**Fig 10:**
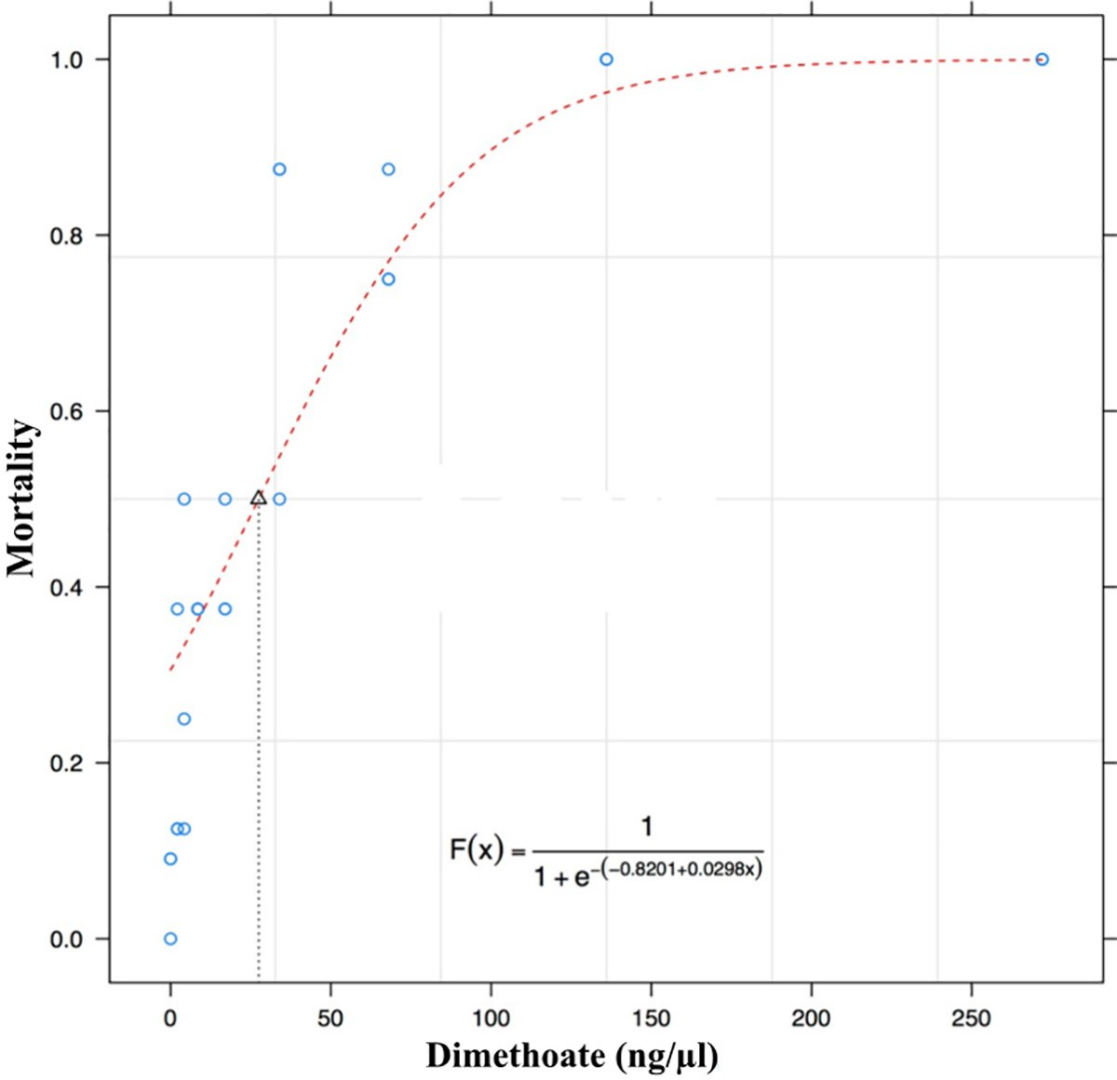
Oral toxicity of the insecticide dimethoate for *Melipona scutellaris* larvae.

Based on the toxicity tests from the insecticide dilution directly on the larval food, the LC_50_ value was 27.48 ng dimethoate / μL of diet, with the lower and upper limits being 19.8 and 35.16 ng / kg of diet, respectively (Table 4 and Fig 10).

**Table 4:** Oral toxicity of insecticide dimethoate for *Melipona scutellaris*.

Time (hours)	LC_50_	CI _95%_	DF	X^2^m
**144**	27.48	19.8–35.16	25	23.5
**168**	27.48	19.8–35.16	25	23.5

LC_50_: mean lethal concentration in ng /μL of diet; CI_95%_: Confidence interval at 95%; FD: freedom degrees; X^2^m: chi-square model.

## Discussion

### Development

The four experiments in this work were performed sequentially and addressed the required need for the proposal of a protocol; they showed no statistically significant difference among the emergence and mortality rates in each experiment and morphometrically resembled the magnitudes of the naturally emerged individuals.

According to OECD [36], which uses the species *A*. *mellifera* (with an already standardized rearing method) as a model organism for toxicity tests, the contaminated diet must be offered to the larvae from the 3^rd^ to the 6^th^ day of the feeding period, observing the cumulative mortality from the 4^th^ to the 8^th^ day, a period corresponding to the phase of progressive feeding of these bees.

This protocol still in the final validation stage, since the method requires a repeated exposure of the larvae to the pesticide. However, if we consider the rates of the method [21], the mortality in the control plates should not exceed 15% among the replicates. This standardization essentially used the study of Aupinel et al. [22], which obtained a maximum relative emergence rate to the larvae of 69.7% and a relative emergence rate to the pupae of 87.34%.

Compared to the OECD protocol and the work of Aupinel et al. [22], the exposure performed with the individuals of *M*. *scutellaris*, that is, the transfer of food and the addition of NaCl at the end of the feeding, produced very satisfactory results that are above those that are established (9.82% of larval mortality, 80.2% of relative emergence to the larvae and 92.61% to the pupae).

In addition, other works already performed with the larvae of stingless bees carried out by Menezes et al. [27] and Rosa et al. [20] obtained an average of 76.4% emergence, which shows that this work has a very positive result and can be considered representative for future applications in bioassays of toxicity studies on stingless bees at the immature stage.

As previously established, during larval transference, it is not possible to distinguish the brood cells in which the workers, queens or males will emerge. In species of the genus Melipona, queens emerge from isomorphic cells in relation to cells occupied by workers or males. The amount of food is the same, and the caste determination system is regulated by genetic mechanisms and by environmental influence (Hartfelder et al., 2006).

Actually, we focused on a rearing protocol, in other words, in the definition of the required techniques such as the collection of the combs, the transport of these to the laboratory, the model of plates for rearing, the temperature and humidity control of the room where the transfers were carried out, inside the BOD, as well as well as from the inner part of the Petri dish where the acrylic plates were kept throughout the rearing process. In relation to the inclusion of males and queens in mortality and emergency calculations, we transferred to an acrylic plate, larvae from the same brood comb. Thus, we reproduced the already established proportions inside the nest, of the different castes that would emerge. In any case, the *in vitro* rearing method that we used provides new possibilities for studies aimed at the specific rearing of isolated castes, perhaps by manipulation in the composition of the food, if there is differentiation between queens, males and workers, for example.

An essential point for the success of *in vitro* rearing is the recommendation of not using eggs for the *in vitro* transfer, as in all cases, the emergence rates have been close to zero. Eggs and larvae need to maintain contact with the air for respiration, which is easily understood in groups of bees that have a progressive supply of larval food, as in *A*. *mellifera* and *Bombus* species as well as in bee groups with a solid food supply. However, in stingless bees, the larval food is liquid, and the position of the egg on the food in this consistency required evolutionary adaptations for this group [37]. According to Velthuis [37], the eggs come into contact with the fluid for only approximately one-seventh of its length and 5% of its volume. Thus, in the present study, manipulation with the needle may have submerged the eggs more than allowed, causing mortality at this stage of development.

The high success in emergence rates was also due to the use of acrylic plates made especially for the execution of these bioassays because it simulated the dimensions of the natural brood cells. Another fundamental point for the success of rearing is the internal control of humidity in the artificial breeding cells, as was already verified by Menezes et al. [27]. According to Camargo [38], in natural conditions, after ingesting all the food, the stingless bee broods weave a cocoon of silk, and they will become pupae. When the broods begin to make the cocoon, the wax and cerumen that constitute the operculum and the bottom of the rearing cells are gradually removed by the workers. As a result, during the feeding period, we kept the humidity at 95 ± 5%, and at the end of the period, we added NaCl, reducing the humidity to approximately 75%.

**Fig 11:**
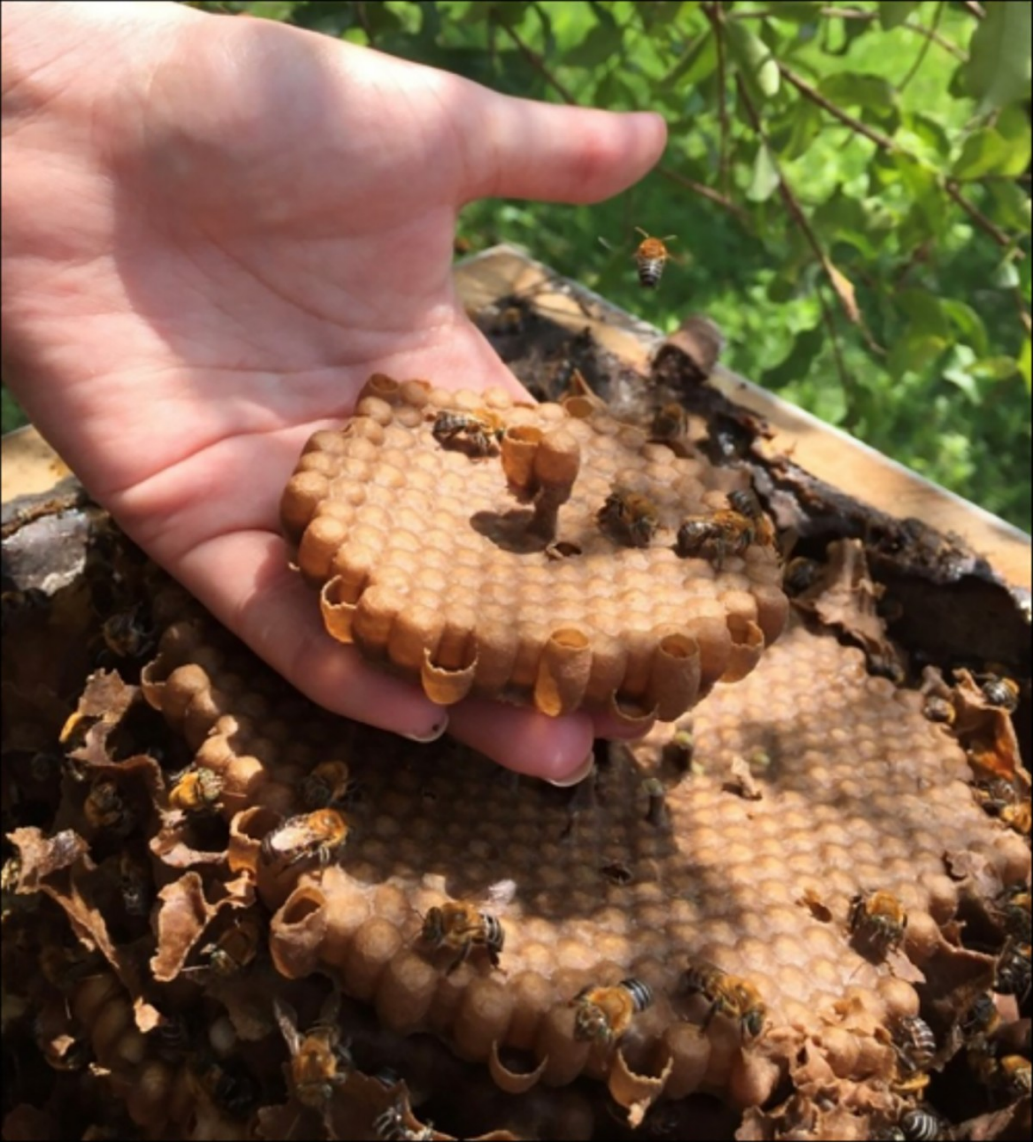
Discoidal form of the breeding of *Melipona scutellaris*.

The minimum differences in the duration of each development phase from the larval transfer were probably due to the transfer of some larvae at approximately 24 hours, which can occur due to the building of the comb, which is performed in a discoidal way with the position of eggs from the inside to out (Fig 11). Thus, the oviposited eggs in the center of the comb will be slightly older than the eggs at the edges of the comb.

### Morphometry

The comparison of the measures of intertegular distance and head width did not show significant differences between bees emerged *in vitro* and *in vivo*. For floating asymmetry, the wing venation pattern indicated a statistically significant difference in the “individual” effect in the *in vitro* treatment, suggesting there may be some level of developmental stress when compared to *in vivo* treatment, influencing the morphology variation. Nevertheless, these variations are expected in individuals in nature, since these variations occur naturally as a function of environmental stress factors, such as pollution, restriction of food availability, etc. [39]. Thus, the occurrence of a statistically significant difference in the “individual” effect in the *in vitro* treatment does not make the method unfeasible. The other parameters and effects evaluated were similar among treatments.

In addition, a study carried out with honey bees showed that individuals reared *in vitro* were not morphologically (structural differences in the wing muscle) identical to those reared in the hive and, even so, this protocol is still widely accepted [40]. This may reinforce robustness of our *in vitro* method.

Thus, the comparative morphometric results generated with *M*. *scutellaris* allow *in vitro* emerged bees to represent bees emerged in *in vivo* bioassays.

### LC_50_ of dimethoate

Dimethoate is the standard insecticide used for the validation of toxicological tests with bees. This molecule was adopted as a reference because it was stable in tests carried out in different places throughout the world, pointing out nonsignificant variations in the lethal concentrations (LCs) and lethal doses (LDs) evaluated [41]. Thus, the OECD [21] requires that all toxicological bioassays with *A*. *mellifera* larvae for pesticide risk assessment be validated by the LC_50_ of dimethoate (lethal concentration that kills 50% of the population) already established for this species (8.8 μg active ingredient (ia) / larva).

In our study, the LC_50_ for *M*. *scutellaris* was 0.02748 μg dimethoate / larvae. Thus, considering CLs_50_, *M*. *scutellaris* is 320 times more sensitive to dimethoate than *A*. *mellifera*.

For toxicological tests, another point must be highlighted. The massive feeding of stingless bees allows the food used in the artificial cells to be withdrawn directly from the natural brood cells, and the diet is collected directly from the brood combs in the day of the larval transfer. Thus, any environmental pesticides that are present in the hive will already be included as a baseline in the diet being offered to the larvae in “controlled” experimental groups. Therefore, we recommend, when it is possible, the sample of the brood food should be retained from each collection for analysis to identify any pesticides already present in the diet. The use of the “High Liquid Perfomance Cromatography” (HPLC) technique did not work for this kind of analysis in our larval food samples. The stingless bee’s larval food is liquid, composed by a mixture of pollen, gland secretions, and honey [19] and, more recently, the presence of fungi was also detected inside brood cells [42]. Thus, probably, the composition and consistence of the stingless bees’ larval food prevented this assessment with HPLC, useful for analysis of several honey bee products. Thus, other techniques should be considered for future analysis of larval food of stingless bees.

## Conclusion

From the tests performed *in vitro* in the present study, we infer the potential nonuse of none of the standardized method for *A*. *mellifera* in stingless bees. In *A*. *mellifera*, there is either single or continuous exposure to the active ingredient and, on the other hand, in *M*. *scutellaris*, there is a joint of both processes, since the deposition of all the food that will be consumed by the larvae occurs only on the first day, being gradually consumed on consecutive days (approximately five days). In other words, in stingless bees occur a single offer of the active ingredient (in the first day) followed by the continuous exposure in consecutive days.

The method described here is of extreme importance for the execution of future toxicological bioassays with larvae of *M*. *scutellaris* and can be adapted to other species of stingless bees.

This work provides evidence of the relevance of the described protocol, showing the difference between this method and the method described for *A*. *mellifera*, thus indicating the importance of tests being standardized for stingless bees due to the differences in their biology and mainly to the differences in their food. In addition, we determined the LC_50_ of dimethoate for *M*. *scutellaris*, which should be used to validate the toxicological bioassays with this species. Another important point to highlight concerns the LC_50_ is that, for *M*. *scutellaris* was found to be 320 times more sensitive than *A*. *mellifera* to the active ingredient dimethoate, which further reinforces the need to develop tests with Brazilian species rather than adopting *A*. *mellifera* as a model.

This proposal is essential because this protocol is the first step for a standardized method and, thereafter, for the validation of a method, according to the OECD, and for it to be considered a validated protocol, several laboratories in different locations should develop the same proposal (ring-tests) and find the same results.

The protocol described in this study for *M*. *scutellaris* showed satisfactory development rates and produced newly emerged workers with similar dimensions to those produced under natural conditions, thus allowing their use in toxicity tests

## Supporting information

S1 Video Combs remove. (MOV)

S2 Video Ecloding egg. (MOV)
