# Correction: *In vitro* larval rearing protocol for the stingless bee species *Melipona scutellaris* for toxicological studies

**DOI:** 10.1371/journal.pone.0223027

**Published:** 2019-09-19

**Authors:** Adna Suelen Dorigo, Annelise de Souza Rosa-Fontana, Hellen Maria Soares-Lima, Juliana Stephanie Galaschi-Teixeira, Roberta Cornélio Ferreira Nocelli, Osmar Malaspina

The first two authors, Adna Suelen Dorigo and Annelise de Souza Rosa-Fontana, should be noted as contributing equally to this work.

The value of LC_50_ appears incorrectly throughout the text. The correct LC_50_ value is 27.48 dimethoate / larvae. The lower and upper limits of the LC_50_ value appears incorrectly in the Results. They should be 19.8 and 35.16 ng dimethoate / larvae, respectively.

In Table 4, the table legend lists the incorrect LC_50_ variable measurement. The correct variable measurement is: LC_50_: mean lethal concentration in ng /larvae. Please see the correct Table 4 here.

**Table 4:** Oral toxicity of insecticide dimethoate for *Melipona scutellaris*.

Time (hours)	LC_50_	CI_95%_	DF	X^2^m
**144**	27.48	19.8–35.16	25	23.5
**168**	27.48	19.8–35.16	25	23.5

LC_50_: mean lethal concentration in ng /larvae. CI_95%_: Confidence interval at 95%; FD: freedom degrees; X^2^m: chi-square model.
